# MicroRNA-301a knockout attenuates peripheral nerve regeneration by delaying Wallerian degeneration

**DOI:** 10.4103/NRR.NRR-D-24-00081

**Published:** 2024-07-29

**Authors:** Lanya Fu, Xiaofang Hu, Jiawei Xu, Zhenlin Li, Jiale Cai, Xinrui Ma, Ying Zou, Ye He, Shuyi Xu, Yizhou Xu, Jiaqi Zhang, Yunlun Li, Jingmin Liu, Tsz Hei Fong, Xianghai Wang, Lixin Zhu, Dongfeng Chen, Aijun Liu, Xiaodong Ma, Jiasong Guo

**Affiliations:** 1 Department of Histology and Embryology, Guangdong Provincial Key Laboratory of Construction and Detection in Tissue Engineering, National Demonstration Center for Experimental Education, School of Basic Medical Sciences, Southern Medical University, Guangzhou, Guangdong Province, China; 2 Key Laboratory of Mental Health of the Ministry of Education, Guangdong-Hong Kong-Macao Greater Bay Area Center for Brain Science and Brain-Inspired Intelligence, Guangdong Province Key Laboratory of Psychiatric Disorders, Guangzhou, Guangdong Province, China; 3 Department of Spine Orthopedics, Zhujiang Hospital, Southern Medical University, Guangzhou, Guangdong Province, China; 4 Research Center of Integrative Medicine, School of Basic Medical Sciences, Guangzhou University of Chinese Medicine, Guangzhou, Guangdong Province, China; 5 Key Laboratory of Brain, Cognition and Education Sciences, Ministry of Education, Guangdong Key Laboratory of Mental Health and Cognitive Science, Center for Studies of Psychological Application, Institute for Brain Research and Rehabilitation, South China Normal University, Guangzhou, Guangdong Province, China

**Keywords:** axonal regeneration, CXCR4, macrophage, migration, miR-301a, peripheral nerve injury, phagocytosis, remyelination, Schwann cell, Wallerian degeneration, YY1

## Abstract

Our recent study demonstrated that knockout of microRNA-301a attenuates migration and phagocytosis in macrophages. Considering that macrophages and Schwann cells synergistically clear the debris of degraded axons and myelin during Wallerian degeneration, which is a prerequisite for nerve regeneration, we hypothesized that microRNA-301a regulates Wallerian degeneration and nerve regeneration via impacts on Schwann cell migration and phagocytosis. Herein, we found low expression of microRNA-301a in intact sciatic nerves, with no impact of the microRNA-301a knockout on nerve structure and function. By contrast, we found significant upregulation of microRNA-301a in injured sciatic nerves. We established a sciatic nerve crush model in microRNA-301a knockout mice, which exhibited attenua9ted morphological and functional regeneration following sciatic nerve crush injury. The microRNA-301a knockout also led to significantly inhibited Wallerian degeneration in an *in vivo* sciatic nerve-transection model and in an *in vitro* nerve explant block model. Schwann cells with the microRNA-301a knockout showed inhibition of phagocytosis and migration, which was reversible under transfection with microRNA-301a mimics. Rescue experiments involving transfection of microRNA-301a-knockout Schwann cells with microRNA-301a mimics or treatment with the C–X–C motif receptor 4 inhibitor WZ811 indicated the mechanistic involvement of the *Yin Yang* 1/C–X–C motif receptor 4 pathway in the role of microRNA-301a. Combined with our previous findings in macrophages, we conclude that microRNA-301a plays a key role in peripheral nerve injury and repair by regulating the migratory and phagocytic capabilities of Schwann cells and macrophages via the *Yin Yang* 1/C–X–C motif receptor 4 pathway.

## Graphical abstract

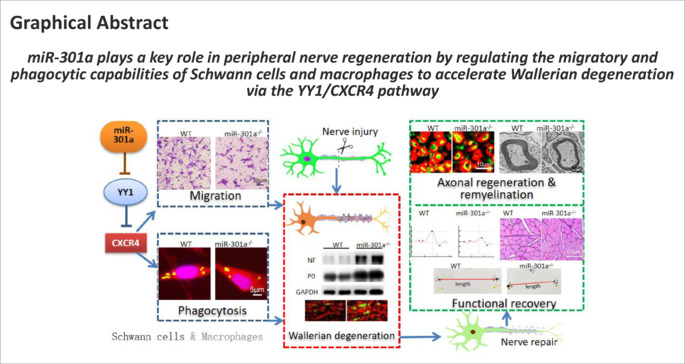

## Introduction

Peripheral nerves are distributed throughout the body, and almost any trauma or surgery inevitably leads to varying degrees of peripheral nerve damage (Vijayavenkataraman, 2020; Lopes et al., 2022; Zhai and Qian, 2024). However, peripheral nerves do have some regenerative capacity, albeit with a slow rate of axonal regrowth (about 1 mm per day), which is the core explanation for the poor efficacy of therapy for peripheral nerve injury (Scheib and Höke, 2013). Consequently, efforts to elucidate the regulatory mechanism of nerve regeneration and find effective new strategies to accelerate nerve regeneration have attracted much attention.

The microRNA (miR)-301a is highly expressed in many tumor cells, playing crucial roles in their proliferation, migration, and invasion, and can be released to other cells through exosomes (Yue et al., 2016; Lan et al., 2018; Wang et al., 2018). Inhibition of miR-301a is now considered a prospective strategy for tumor therapy, but the applicability of this strategy in peripheral nerve regeneration is unknown. Our recent study demonstrated that miR-301a deficiency weakens the migratory and phagocytic capabilities of macrophages (Xu et al., 2022). Phagocytic macrophages and Schwann cells clear the debris of disintegrated axons and myelin during Wallerian degeneration (WD) of injured peripheral nerves (Li et al., 2020; Feng et al., 2023; Ding et al., 2024). Furthermore, WD has been shown to be a prerequisite for subsequent nerve regeneration (Gomez-Sanchez et al., 2017; Xu et al., 2021). Therefore, we hypothesized that miR-301a might also play important roles in Schwann cell biofunctions and WD progression, thereby affecting peripheral nerve regeneration.

To explore this hypothesis, the present study utilized miR-301a knockout (KO) mice to develop three peripheral nerve injury models: sciatic nerve crush, sciatic nerve transection, and nerve explant culture. The effects of miR-301a KO on axonal regeneration, remyelination, functional recovery of motor function, nerve conduction, myoatrophy, and WD were investigated. Finally, the underlying mechanism of miR-301a in nerve injury and repair was assessed using primary cultured Schwann cells and nerve injury models.

## Methods

### Animals

To avoid the influence of the menstrual cycle on the experimental results, only male animals were used in this study. Briefly, specific-pathogen-free C57BL/6J adult mice (age: 8 weeks; body weight: 20–22 g; 81 wild type (WT) and 69 miR-301a KO) and neonatal mice (age: 3–4 days; 59 WT and 51 miR-301a KO) were provided by the Animal Center of Southern Medical University (license No. SCXK (Yue) 2016-0041). The generation and genotyping of miR-301a-KO mice in a C57BL/6J background were performed as previously described (Ma et al., 2015; Xu et al., 2022). The study was performed following the approval of the Institutional Animal Care and Use Committee of Southern Medical University (approval No. SMU-L2021183) in June 2021. All experiments were designed and reported in accordance with the Animal Research: Reporting of *In Vivo* Experiments (ARRIVE) guidelines (Percie du Sert et al., 2020). Every effort was made to minimize the usage of animals and their suffering.

### Preparation of nerve injury models

#### Sciatic nerve crush injury

Adult mice were anesthetized by intraperitoneal injection of 12 mg/mL tribromoethanol (Sigma-Aldrich, St. Louis, MO, USA) at a dose of 180 mg/kg body weight. The left sciatic nerves were bluntly exposed and crushed at 0.5 cm distal to the sciatic notch by clamping the nerve with a smooth, straight hemostat for 2 minutes, as described in our previous reports (Hu et al., 2020; Liu et al., 2023). The distal trunk of the lesion nerve was harvested at 0, 7, 14, and 28 days post-crush injury (dpi) to assess the miR-301a expression level. From 25 to 27 dpi, the mice underwent behavioral test training three times per day. At 28 dpi, the mice were subjected to formal behavioral tests, followed by electrophysiology and tissue collection for further experiments, as described previously (Liu et al., 2023).

#### Sciatic nerve transection injury

In this model, the procedures were the same as those for the nerve crush injury model, except that the left sciatic nerves were cut at 0.5 cm distal to the sciatic notch. The nerve-transected mice that survived for 5 days were then subjected to WD assays.

#### Nerve explant culture

**Figure 1:**
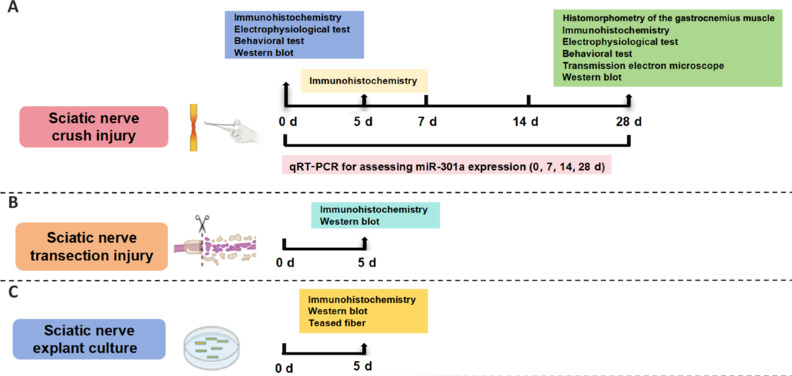
Schematic diagrams showing the design of the major study experiments. (A) Sciatic nerve crush injury model used to explore the expression of miR-301a over time, the migration of Schwann cells in the injured sciatic nerve at 5 days post crush injury, and the nerve regeneration and functional recovery of mice at 28 days post crush injury. The morphological structure and function of intact nerves were examined prior to the injury. (B) Sciatic nerve transection injury model used to evaluate Wallerian degeneration *in vivo* via detection of the remaining axons and myelin in the nerve at 5 days post transection injury. (C) Sciatic nerve explant culture designed to confirm the Wallerian degeneration via detection of the remaining axons and myelin in the nerve after 5 days of nerve segment culture *in vitro*. miR-301Aa: MicroRNA-301a.

Bilateral sciatic nerves dissected from anesthetized adult mice were cut into 3-mm-long segments and cultured for 5 days, as described in our previous reports (Liu et al., 2022a; Zou et al., 2022). The explants were then collected for immunohistochemistry or western blotting. Additionally, some explants were teased into single-nerve fibers and mounted on slides for the assessment of myelin ovoid formation following published protocols (Wen et al., 2017; Liu et al., 2022b). The experimental design of the study is shown in **Figure 1**.

### Behavioral tests of nerve-injured mice

#### Gait analysis

For assessment of walking function after sciatic crush injury (Matias Júnior et al., 2019; Yin et al., 2022), mice were trained to walk on a glass plate in an enclosed walkway. The ipsilateral footprints of injured mice were analyzed to calculate the stride length (distance between the front and rear paws) and the lateral rotation angle (angle between the rear paw and its forward direction), as described previously (Li et al., 2019a).

#### Rotarod test

Twelve hours after the gait analysis, motor coordination of sciatic crush-injured mice was quantitatively evaluated (Lubrich et al., 2022; Cheung et al., 2023) by placing the mice on a rotarod apparatus (YLS-4C, Jinan Yiyan Technology, Jinan, China) at accelerating speed (5–40 revolutions/minute [rpm] over 90 seconds) or fixed speed (40 rpm). The duration from beginning to fall-down was recorded using the YLS-4C apparatus software (Brooks and Dunnett, 2009; Pan et al., 2017).

### Electrophysiological test

Two hours after the rotarod test, the mice were anesthetized with tribromoethanol as described above. A pair of stimulating electrodes were applied to the sciatic nerve 3 mm proximal to the injury site and a pair of recording electrodes were placed in the intrinsic foot muscles (Pan et al., 2017; Hu et al., 2020) for measurement of the amplitude and latency of the compound muscle action potential using an electrophysiological system (Axon Digidata 1550 Digitizer, Molecular Devices, Sunnyvale, CA, USA).

### Tissue collection

For histological staining, mice were anesthetized with an intraperitoneal injection of an overdose of 12 mg/mL tribromoethanol as described above, sacrificed, and intracardially perfused with 4% paraformaldehyde (PFA, Aladdin, Shanghai, China). For transmission electron microscopy, the mice were anesthetized as above, sciatic nerve and gastrocnemius muscle were harvested and postfixed in 4% PFA overnight to prepare cryosections or teased fiber preparations. Some of the sciatic nerve segments were postfixed in a 2.5% solution of glutaraldehyde in 2% PFA (Nacalai Tesque Inc, Kyoto, Japan). For western blotting, mice were decapitated prior to the preparation of protein lysates from a 7-mm nerve segment isolated from the distal lesion site.

### Immunohistochemistry

Harvested nerve segments and cultured nerve explants were post-fixed in 4% PFA overnight and then infiltrated with a series of sucrose solutions before being embedded in optimum cutting temperature compound (OCT 4583, Sakura, Torrance, CA, USA). The frozen tissues were sliced into 10-μm sections on a cryostat (Leica CM1950, Wetzlar, Germany). After rinsed with phosphate-buffered saline (PBS), the sections were permeabilized with 0.5% Triton X-100 (Sigma, St. Louis, MO, USA) for 30 minutes, then blocked with 5% fish gelatin (Cat# G7041, Sigma) containing 0.3% Triton X-100 at room temperature (25–27°C) for 1 hour. Next, the sections were incubated with primary antibodies at 4°C overnight and then with secondary antibodies for 2 hours at room temperature. Finally, all sections were incubated with 1 µg/mL 4′,6-diamidino-2-phenylindole (DAPI, 1:1000 dilution; Cat# D8417, Sigma) for 2 minutes to counterstain cell nuclei. The antibody details are provided in **Additional Table 1**.

**Additional Table 1:** Antibody list

Antibody	Animal species	Dilution	Supplier	Cat#	RRID
NF200	Mouse	1:2000 (WB) 1:200 (IF)	Sigma, St. Louis, MO, USA	N5389	AB_260781
P0	Rabbit	1:2000 (WB) 1:200 (IF)	Millipore, Burlington, MA, USA	ABN363	AB_2936982
CNPase	Rabbit	1:200 (IF)	Bioword Technology, Louis Park, MN, USA	BS3461	AB_1662288
S100	Rabbit	1:200 (IF)	Sigma	SAB4502708	AB_10744555
Laminin	Rabbit	1:200 (IF)	Abcam, Cambridge, UK	ab7463	AB_305933
YY1	Rabbit	1:2000 (WB)	Abcam	ab109237	AB_10890662
P75	Rabbit	1:200 (IF)	Abcam	ab52987	AB_881682
CXCR4	Rabbit	1:2000 (WB)	Abcam	ab181020	AB_2910168
GAPDH	Rabbit	1:1000 (WB)	Abcam	ab128915	AB_11143050
Tuj1	Mouse	1:400 (IF)	Sigma	T8660	AB_477590
HRP-conjugated secondary antibody	Rabbit	1:2000 (WB)	Molecular Probes, Eugene, OR, USA	65-6120	AB_2533967
HRP-conjugated secondary antibody	Mouse	1:2000 (WB)	Invitrogen, Carlsbad, CA, USA	SA5-10317	AB_2868364
Goat anti-rabbit Alexa Fluor 568	Goat	1:400 (IF)	Invitrogen	A-11011	AB_143157
Goat anti-mouse Alexa Fluor 488	Goat	1:400 (IF)	Invitrogen	A-21121	AB_2535764
Goat anti-rabbit Alexa Fluor 488	Goat	1:400 (IF)	Invitrogen	A-11008	AB_143165
Goat anti-mouse Alexa Fluor 568	Goat	1:400 (IF)	Invitrogen	A-11004	AB_2534072

CXCR4: C-X-C motif receptor 4; GAPDH: glyceraldehyde-3-phosphate dehydrogenase; HRP: horseradish peroxidase; IF: immunofluorescence; NF200: neurofilament 200; P0: myelin protein zero; Tujl: β-tubulin III; WB: Western blotting; YY1: Ying-Yangl.

### Histomorphometry of the gastrocnemius muscle

The harvested gastrocnemius muscle (*n* = 6 for each group) was weighed to calculate the wet weight ratio of the ipsilateral muscle to that of the contralateral side (Pan et al., 2017). Next, the mid-belly of the muscle was transversally sectioned and subjected to routine staining with hematoxylin-eosin (He et al., 2017) or immunostaining with rabbit anti-laminin antibody (1:200 dilution; Abcam, Cat# ab7463) to visualize the myofibers. Five random non-overlapping fields of five sections from each animal were captured, and the area of myofibers was quantified using ImageJ software 6.0 (National Institutes of Health, Bethesda, MD, USA), as described in a previous study (Xu et al., 2021).

### Transmission electron microscopy

Distal nerve segments (*n* = 3 for each group) between 3 and 5 mm from the lesion site were postfixed in glutaraldehyde and osmium tetroxide, dehydrated in graded acetone, embedded in Spurr’s resin (Sigma-Aldrich), and sliced into ultrathin (70 nm) cross sections. After staining with uranyl acetate and lead citrate, five images of each section were captured with a transmission electron microscope (H-7500, Hitachi, Tokyo, Japan) and loaded into ImageJ software to measure the diameters of nerve fibers and axons. The g-ratio of the myelinated nerve fiber was calculated by dividing the axon diameter by the nerve fiber diameter (Liu et al., 2023).

### Oil red O and fluoromyelin staining

To illustrate the degraded and remaining myelin, transverse and longitudinal sections of the injured nerves were stained with oil red O (ORO) (Cat# G1262, Solarbio, Beijing, China,) and fluoromyelin^TM^ (1:2000 dilution; Cat# F34652, Thermo Fisher, Waltham, MA, USA). Some of the ORO-stained sections were counterstained with S100 immunofluorescence as described above to evaluate the engulfment of myelin debris in Schwann cells (Liu et al., 2022a; Zou et al., 2022). After routine immunostaining, the sections were incubated in the mixture of 0.3% ORO (in 60% 2-propanol) and deionized water (ratio 3:2) for 15 minutes and then rinsed in 60% isopropanol and 0.01 M PBS. For fluoromyelin staining, the nerve sections were incubated with fluoromyelin for 1 hour at room temperature. The mean fluorescence intensity of ORO in each S100-positive cell, as well as the mean fluorescence intensity of ORO or fluoromyelin in each field, were counted using ImageJ software.

### Dorsal root ganglion neuron culture

Following our previous protocols (Lim et al., 2017; Tan et al., 2020), neonatal (postnatal days 3) mice were sacrificed after anesthetization by hypothermia (Phifer and Terry, 1986; No authors listed, 2015). The L4–L6 dorsal root ganglion (DRG) was dissected, digested with 0.25% trypsin-ethylenediaminetetraacetic acid (EDTA; Cat# 25200072, Gibco, Grand Island, NY, USA) for 30 minutes, gently triturated 30 times, resuspended in Dulbecco’s modified Eagle’s medium/F12 (DMEM/F12, Cat# 11330057, Gibco) containing 3% fetal bovine serum (FBS, Cat# BC-SE-FBS01C, BioChannel Biological Technology, Nanjing, China), and cultured on glass coverslips (Fisher Scientific, Pittsburgh, PA, USA) coated with poly-l-lysine (Cat# P1274, Sigma-Aldrich) for 24 or 72 hours. The cultures were then fixed in 4% PFA for 30 minutes and subjected to immunostaining with mouse anti-β-tubulin III (Tuj1, 1:400 dilution; Sigma-Aldrich, Cat# T8660), as described in the Immunohistochemistry section above.

### Schwann cell culture

Schwann cells were isolated from postnatal day 3 mice as previously described (Wen et al., 2018; Liu et al., 2022a). Briefly, the neonatal mice were anesthetized by hypothermia, the sciatic and spinal nerves were collected and digested with 0.125% Trypsin-EDTA for 30 minutes, and the isolated cells were resuspended in DMEM/F12 containing 10% FBS and plated onto poly-l-lysine-coated petri dishes (Jet Biofil, Guangzhou, China). Twenty-four hours later, the cells were treated with 10 μM cytosine arabinoside (Cat# 147-94-4, Sigma-Aldrich) for 48 hours to eliminate fibroblasts. The cells were then cultured in DMEM/F12 containing 10% FBS, 3 μM forskolin (Cat# F6886-10MG, Sigma-Aldrich), 4 mM L-glutamine (Cat# A29168-01, Gibco), and 10 ng/mL heregulin (Cat# 100-03, PeproTech, Cranbury, NJ, USA). The Schwann cells were identified by immunostaining with antibodies against S100 (1:200 dilution; Sigma, Cat# SAB4502708), P75 (1:200 dilution; Abcam, ab52987), and CNpase (1:200 dilution; Bioword Technology, Cat# BS3461; Wen et al., 2017; Xue et al., 2023a).

### miR-301a mimics transfection and WZ811 treatment of cultured Schwann cells

To overexpress miR-301a in Schwann cells, 3rd-passage Schwann cells were transfected with miR-301a mimics or miRNA negative control (NC) vectors (RiboBio, Guangzhou, China) at 50 nM, in accordance with the manufacturer’s instructions. Two days later, the transfection medium was removed and the cells were cultured in Schwann cell culture medium as described above for further 24 hours.

To verify the role of chemokine C–X–C motif receptor 4 (CXCR4) in Schwann cell biofunctions, miR-301a mimics- or miRNA NC-transfected Schwann cell cultures were treated for 48 hours with 10 μM WZ811 (Selleck, Houston, TX, USA), a CXCR4 inhibitor, prior to the migration and phagocytosis tests.

### Phagocytosis assay

Schwann cells were supplemented with 0.1 mg/mL fluorescent lumispheres (1 µm-diameter; BaseLine Chromtech, Tianjin, China) for 8 hours. After three rinses with PBS, the cultures were fixed with 4% PFA, immunostained with anti-S100 antibody, and the number of lumispheres engulfed by each cell was counted (Xu et al., 2022).

### Transwell migration assay

The migration of cultured Schwann cells was examined using Transwell chambers (8-µm pores, Cat# 3422, Corning Costar, New York, NY, USA), as described previously (Wen et al., 2017). A total of 1 × 10^5^ Schwann cells resuspended in 200 µL DMEM/F12 containing 1% FBS were seeded into the upper chamber, while the lower chamber was filled with 600 µL DMEM/F12 containing 10% FBS. After 20 hours, the chambers were fixed with 4% PFA for 30 minutes. The cells on the upper surface were carefully removed with a cotton swab, while those adhering to the lower surface of the Transwell membrane were stained with 0.1% crystal violet (Leagene, Beijing, China, DZ0055) for 40 minutes. Five images of each membrane (the center and four quadrants) were captured under a fluorescent microscope (Leica CM1950, Wetzlar, Germany) for quantification.

### Western blotting

Nerve tissues and Schwann cells were lysed in radioimmunoprecipitation assay lysis buffer (Cat# FD009, Fdbio Science, Hangzhou, China) containing a protease inhibitor cocktail (1:100 dilution; Cat# FD1001, Fdbio Science). The protein lysates were separated by sodium dodecyl sulfate-polyacrylamide gel electrophoresis (Cat# FD006, Fdbio Science) and transferred to a polyvinylidene difluoride membrane (Cat# IPVH0010, Millipore, Burlington, MA, USA). The blots were blocked with 5% bovine serum albumin for 1 hour, incubated with primary antibodies overnight at 4°C, and then incubated with horseradish peroxidase-conjugated secondary antibodies at room temperature for 2 hours. The antibody details are provided in **Additional Table 1**. After visualization using enhanced chemiluminescence (Millipore), the blot images were captured by a Lumazone system (Roper, Trenton, NJ, USA), the integrated optical density of each blot was quantified using ImageJ software, and the relative protein levels were determined by normalization to the band intensity of glyceraldehyde-3-phosphate dehydrogenase.

### Quantitative reverse transcription polymerase chain reaction

Total RNA was extracted from crush-injured sciatic nerves at 0, 7, 14, and 28 dpi using TRIzol buffer (Tiangen, Beijing, China). The expression of miR-301a was examined using the Bulge-Loop miRNA quantitative reverse transcription PCR (qRT-PCR) Starter Kit (Ribobio), with the small nuclear RNA U6 assigned as the internal standard. The primer sequence used to amplify miR-301a was 5′-CAG UGC AAU AGU AUU GUC AAA GC-3′. Relative quantification of miR-301a expression was performed using the 2^–ΔΔCT^ method (Mestdagh et al., 2008; Shen et al., 2022; Xue et al., 2023b), where ΔΔCT = ΔCT of the sample – ΔCT of the untreated control. The qRT-PCR parameters were set up in accordance with the instructions of the qRT-PCR Starter Kit.

### Statistical analysis

No statistical methods were used to predetermine sample sizes; however, we did use sample sizes that were similar to those reported in previous publications (Li et al., 2020; Liu et al., 2022). For normally distributed data with equal variance, the statistical analyses were performed with GraphPrism 9.0 (GraphPad Software, Boston, MA, USA, www.graphpad.com) using one-way analysis of variance followed by Bonferroni’s multiple comparison test. The two-tailed Student’s *t*-test for normally distributed data with comparable variances was used to highlight statistical differences between two groups.

## Results

### Knockout of microRNA-301a, which shows increased expression in the injured sciatic nerve, does not affect the morphology and function of the intact nerve

**Figure 2:**
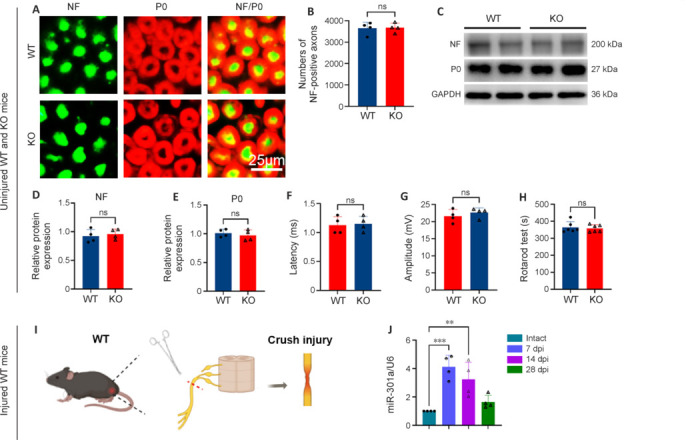
Similar nerve structure, conduction, and motor function profiles in uninjured adult WT and miR-301a KO mice, with upregulated miR-301a expression in the crush-injured sciatic nerve of WT mice. (A, B) Representative immunofluorescence images (A) and quantification (B) showing similar numbers of neurofilament (NF)200-positive axons (green, Alexa Fluor 488) in cross-sections of intact sciatic nerves of WT and miR-301a KO mice (*n* = 4 per group, two-tailed Student’s *t*-test). (C–E) Western blotting (C) and quantification (D, E) illustrating the similar protein levels of NF200 and myelin protein zero (P0) in intact sciatic nerves of WT and KO mice (*n* = 4, two-tailed Student’s *t*-test). (F, G) Compound muscle action potential assessment showing similar latency and amplitude of the intact sciatic nerve of WT mice and KO mice (*n* = 4, two-tailed Student’s *t*-test). (H) Rotarod assessment of motor coordination at constant rotation (40 revolutions/minute; *n* = 6, two-tailed Student’s *t*-test). (I) Illustration of the sciatic nerve crush injury model (created with BioRender.com). (J) Quantitative real-time PCR assay of WT mice showing the miR-301a expression level in distal sciatic nerve segments at 0, 7, 14 and 28 days post-injury (dpi; *n* = 4, one-way analysis of variance followed by Bonferroni’s multiple comparison test). The 2^–ΔΔCT^ values were normalized to the expression of U6. Data are expressed as means ± standard deviation; **P < 0.01, ***P < 0.001. GAPDH: Glyceraldehyde-3-phosphate dehydrogenase; KO: knockout; miR-301a: microRNA-301a; ns: not significant; WT: wild-type.

Considering our plan to utilize miR-301a-KO mice to explore the role of this miR in nerve regeneration, we sought to first investigate the potential effect of miR-301a KO on morphological and functional profiles of mice prior to nerve injury surgery. Immunofluorescence staining revealed that the densities of neurofilament (NF)200-positive axons and protein zero (P0)-positive myelin in intact sciatic nerves were not markedly different between the KO and WT groups (**Figure 2A** and **B**). Western blotting also showed similar levels of NF200 and P0 total protein between the two groups (**Figure 2C–E**). In addition, the compound muscle action potential (**Figure 2F** and **G**) and rotarod assessment (**Figure 2H**) indicated that the miR-301a KO did not affect nerve conduction or motor function in intact WT and KO mice. Furthermore, qRT-PCR analysis of miR-301a in WT mice showed low expression in the intact nerve, followed by marked upregulation at 7 days following sciatic nerve crush injury and a gradual decline thereafter (**Figure 2I** and **J**).

### MicroRNA-301a knockout delays post-injury sciatic nerve regeneration

**Figure 3:**
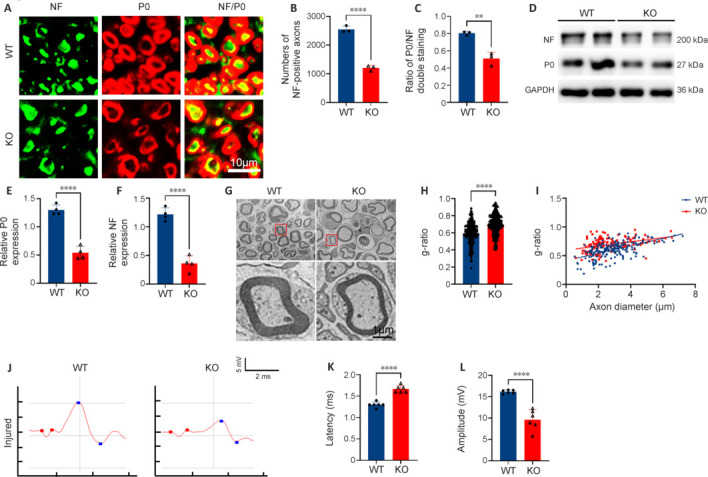
miR-301a deficiency attenuates axonal regeneration and remyelination, as well as nerve conduction, in crushed sciatic nerves. All data were collected from the distal trunk of crush-injured sciatic nerves at 28 days post-injury. (A–C) Representative immunofluorescence images (A) and quantitative analysis (B, C) showing increases in the number of neurofilament (NF)200-positive axons (green, Alexa Fluor 488) and ratio of myelin protein zero (P0)/NF200-positive myelin (red, Alexa Fluor 568) in cross-sections of injured sciatic nerves in wild-type (WT) mice compared with those in miR-301a knockout (KO) mice (*n* = 3). (D–F) Western blotting (D) and quantification (E, F) of P0 and NF200 protein levels showing marked elevations in the WT group compared with those in the KO group (*n* = 4). (G–I) Representative transmission electron microscopy images (G) and calculated g-ratios (H) and the correlation between g-ratio and axon diameter (I), revealing better nerve regeneration in the WT group compared with that in the KO group (*n* = 3). (J) Representative images of compound muscle action potential (CMAP) assessment of the injured sciatic nerve in the two groups. Red dots represent latency; blue dots represent amplitude. (K, L) CMAP assessment showing longer latency (K) and lower amplitude (L) in the KO group compared with that in the WT group (*n* = 6). Data expressed as means ± standard deviation; **P < 0.01, ****P < 0.0001 (two-tailed Student’s *t*-test). GAPDH: Glyceraldehyde-3-phosphate dehydrogenase; miR-301a: microRNA-301a.

In view of the dramatic upregulation of miR-301a in the injured nerve, which suggested that it may be involved in the regulation of nerve injury response and repair, we examined the structural and functional repair at 28 dpi in mice with sciatic nerve crush injury. Immunofluorescence in the KO group revealed a significantly lower density of NF200-positive axons in the distal trunk of the injured nerve, and a markedly reduced percentage of P0/NF200 double-positive nerve fibers in all NF200 single-positive axons, compared with the WT group (**Figure 3A–C**). Collectively, these findings suggested that miR-301a depletion attenuates axonal regeneration and remyelination in the injured nerve. This view was supported by the results of western blotting (**Figure 3D–F**) and transmission electron microscopy (**Figure 3G**). Specifically, g-ratio analysis based on transmission electron microscopy images suggested that the myelin of regenerated nerve fibers was significantly thinner in the KO group than in the WT group (**Figure 3H** and **I**). The link between conduction function and the structural repair of injured nerves is well established. Therefore, we conducted an electrophysiological assessment of compound muscle action potential of the injured nerve (**Figure 3J**), which showed lower amplitude and increased latency in the KO group compared with that in the WT group (**Figure 3K** and **L**).

### MicroRNA-301a knockout impairs functional recovery after sciatic nerve injury

**Figure 4:**
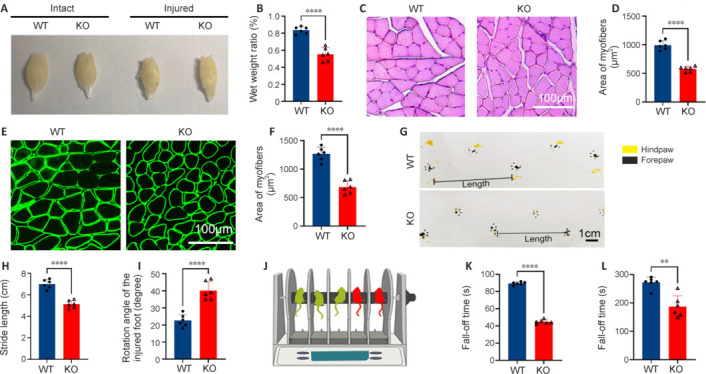
miR-301a deficiency worsens gastrocnemius muscle atrophy and lowers rotarod test scores in sciatic nerve crush–injured mice. (A, B) Glossy images of intact and injured gastrocnemius muscle (A) and quantification of the wet weight ratio of the ipsilateral to contralateral gastrocnemius muscle (B) in miR-301a knockout (KO) and wild-type (WT) mice at 28 days post-injury (dpi) (*n* = 6). (C–F) Representative H&E staining (C) and laminin immunostaining (E) of transverse sections of the gastrocnemius muscle and the quantification of myofibers (D, F), showing a remarkably larger myofiber area in the WT group compared with that in the KO group at 28 dpi (*n* = 6). (G–I) Gait analysis of the two groups (G) and calculations showing a much longer stride length of the injured hindlimb and the ipsilateral forelimb in WT mice (H) and a much larger lateral rotation angle of the injured foot (I) in KO mice at 28 dpi (*n* = 6). (J–L) Illustration of the rotarod test administered to both groups (J), in which the KO group showed significantly shorter fall-off times under both accelerating (K) and constant rotation (L) conditions at 28 dpi (*n* = 6). Data expressed as means ± standard deviation; **P < 0.01, ****P < 0.0001 (two-tailed Student’s *t*-test). H&E: Hematoxylin-eosin; miR-301a: microRNA-301a.

One of the main consequences of nerve injury – myoatrophy in the target muscles – can be reversed to some extent by axonal regeneration and reinnervation of the target muscles. Motor function is also restored to a certain level (Xu et al., 2021). We found that the wet weight ratio of the ipsilateral to the contralateral gastrocnemius muscle was dramatically lower in the KO group than in the WT group (**Figure 4A** and **B**). Furthermore, the myofiber size, as determined by hematoxylin-eosin staining (**Figure 4C** and **D**) and laminin immunofluorescence (**Figure 4E** and **F**), was significantly reduced in the KO group compared with that WT group. Consistently, the gait test demonstrated that KO of miR-301a shortened the stride length and increased the lateral rotation angle of the nerve injury–affected foot (**Figure 4G–I**), while the rotarod test showed that miR-301a-KO mice had a shorter fall-down time compared with WT mice (**Figure 4J–L**). Together, these findings indicated that miR-301a depletion weakens functional repair following sciatic nerve injury.

### Neurite growth of dorsal root ganglion neurons is not affected by the microRNA-301a knockout

**Figure 5:**
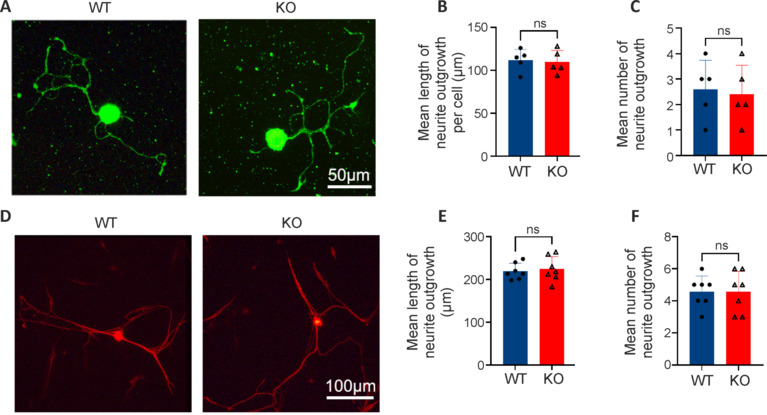
Similar neurite growth of DRG neurons in the miR-301a KO and WT groups. (A, D) Representative immunostaining of anti-β-tubulin III (Tuj1) in dorsal root ganglia (DRG) neurons isolated from WT and miR-301a KO mice and cultured *in vitro* for 24 hours (A; green, Alexa Fluor 488) (*n* = 5) or 72 hours (D; red, Alexa Fluor 568) (*n* = 7). Quantification of the total length (B, E) and number (C, F) of neurites, showing no between-group differences at either time point. Data expressed as means ± standard deviation (two-tailed Student’s *t*-test). KO: Knockout; miR-301a: microRNA-301a; ns: not significant; WT: wild-type.

To clarify whether the above outcomes were related to the involvement of miR-301a in the neurite regeneration ability of neurons, we cultured DRG neurons isolated from WT and KO mice. There were no statistical differences between the WT and KO group neurons in terms of the number of processes or total length of all processes of each neuron following 24 hours (**Figure 5A–C**) or 72 hours (**Figure 5D–F**) of culture *in vitro*.

### MicroRNA-301a knockout slows down the process of Wallerian degeneration

**Figure 6:**
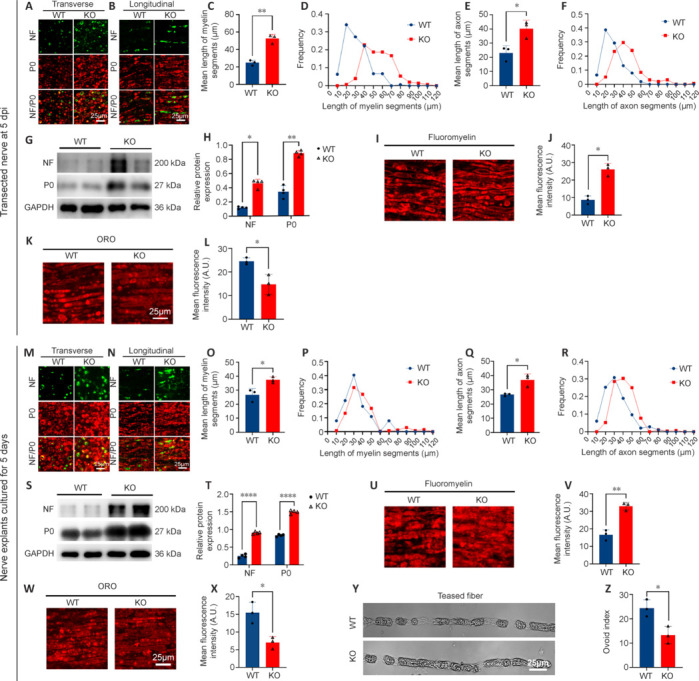
miR-301a deficiency retards Wallerian degeneration in transection-injured nerves *in vivo* and in nerve explant cultures *in vitro*. (A–F) Representative immunofluorescence images showing the segmentation of residual neurofilament (NF) 200-positive axon (green, Alexa Fluor 488) and myelin protein zero (P0)-positive myelin (red, Alexa Fluor 568) segments in transverse (A) and longitudinal (B) sections of the transection-injured nerve at 5 days post-injury (dpi) in wild-type (WT) and miR-301a knockout (KO) mice, with quantification showing significantly shorter lengths of P0-positive myelin (C, D) and residual NF-positive axon (E, F) segments in WT mice (*n* = 3). (G, H) Western blotting (G) and quantification (H) showing lower protein levels of NF and P0 in the transected nerve of WT mice compared with those in KO mice at 5 dpi (*n* = 4). (I–L) Fluoromyelin (I) and oil red O (ORO; K) staining and quantification analyses (J, L) showing significantly less residual myelin and significantly more degraded myelin in the transection-injured nerve of the WT group compared with that of the KO group at 5 dpi (*n* = 3). (M–R) Representative immunofluorescence images of transverse (M) and longitudinal (N) sections of nerve explants cultured for 5 days, with quantitative analyses showing significantly greater segmentation of P0-positive myelin (red; O, P) and residual NF200-positive axons (green; Q, R) in the WT group compared with that in the KO group (*n* = 3). (S, T) Western blotting (S) and quantification (T) showing the protein levels of NF and P0 in the nerve explants of both groups after 5 days of culture (*n* = 4). (U–X) Fluoromyelin (U) and ORO (W) staining and quantification analyses (V, X) of nerve explants cultured for 5 days, showing significantly less residual myelin and significantly more degraded myelin in the WT group compared with that in the KO group (*n* = 3). (Y, Z) Representative images of teased single nerve fibers of the nerve explants from the two groups (Y), with quantitative analysis (Z) showing a significantly higher myelin ovoid index (number of ovoids on nerve fibers of 200 μm in length) in the WT group (*n* = 3). Data are expressed as means ± standard deviation; *P < 0.05, **P < 0.01, ****P < 0.0001 (two-tailed Student’s *t*-test used for all data). GAPDH: Glyceraldehyde-3-phosphate dehydrogenase; miR-301a: microRNA-301a; ns: not significant.

Having shown that KO of miR-301a did not impact the neurite growth of DRG neurons, we hypothesized that the absence of miR-301a in the local microenvironment of the injured nerve might contribute to a delay in nerve regeneration. WD is a main event in the early stages of nerve injury, and is also a prerequisite for creating an environment favorable to nerve regeneration (Yuan et al., 2022). Recently, we reported that miR-301a deficiency weakens the migration and phagocytosis of macrophages (Xu et al., 2022), which led us to hypothesize that miR-301a KO might affect WD. To test this hypothesis in an *in vivo* model with minimal interruption of nerve regeneration, we assessed WD in terms of residual axon and myelin using mice with sciatic nerve transection. Transverse sections (**Figure 6A**) and longitudinal sections (**Figure 6B**) of the transected nerve at 5 dpi showed that the remaining P0-positive myelin segments (**Figure 6C** and **D**) and NF200-positive axonal segments (**Figure 6E** and **F**) were significantly longer in the KO group than in the WT group. The pattern of NF200 and P0 protein levels revealed by western blotting was similar to that under immunostaining (**Figure 6G** and **H**). Furthermore, the intensity of fluoromyelin-labeled residual myelin was higher in the KO group (**Figure 6I** and **J**), while the intensity of ORO staining of degraded myelin was weaker in the KO group (**Figure 6K** and **L**), compared with those in the WT group. Notably, similar results were observed in a nerve explant model after 5 days of culture *in vitro* (**Figure 6M–X**). Moreover, there were far fewer myelin ovoids in teased single nerve fibers from the KO group than in those from the WT group (**Figure 6Y** and **Z**). Overall, these data pointed to a mechanism of delayed WD in miR-301a KO mice.

### Attenuated migration of Schwann cells with microRNA-301a knockout is restored by microRNA-301a mimics transfection

**Figure 7:**
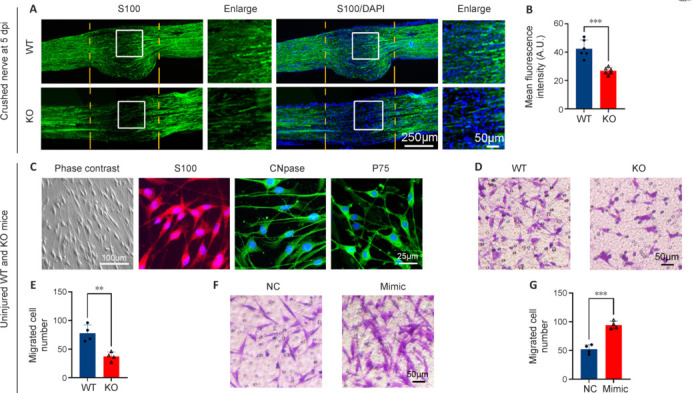
Schwann cell migration is reduced by miR-301a deficiency and enhanced by transfection with miR-301a mimics. (A, B) Immunofluorescence and quantification of S100-positive (green, Alexa Fluor 488) Schwann cell migration from both sides into the epicenter of the crush-injured nerve at 5 days post-injury (dpi), showing significantly less migration in the miR-301a knockout (KO) group compared with that in the wild-type (WT) group (*n* = 3, two-tailed Student’s *t*-test). Counterstaining of nuclei with 4′,6-diamidino-2-phenylindole (DAPI) is also shown. (C) Representative images of phase contrast microscopy and immunofluorescence of primary cultures of Schwann cells isolated from WT mice, showing positivity for the Schwann cell markers S100 (red, Alexa Fluor 568), CNpase (green, Alexa Fluor 488), and P75 (green, Alexa Fluor 488), with DAPI counterstaining (*n* = 3). (D, E) Transwell assay images (D) and quantification (E), showing significantly fewer migrated Schwann cells in the KO group than in the WT group (*n* = 4, two-tailed Student’s *t*-test). (F, G) Transwell assay images (F) and quantification (G) showing significantly more migrated Schwann cells in the miR-301a mimics transfection (Mimic) group than in the negative control miRNA transfection (NC) group (*n* = 4, two-tailed Student’s *t*-test). Data are expressed as means ± standard deviation; **P < 0.01, ***P < 0.001. miR-301a: MicroRNA-301a.

Based on a previous study of Schwann cell activities during peripheral nerve regeneration (Shen et al. 2022), we evaluated Schwann cell migration in WT and miR-301a-KO mice using S100 immunostaining of longitudinal sections of crush-injured sciatic nerves at 5 dpi. Our data indicated that miR-301a deficiency reduced migration of Schwann cells from the two sides into the injured area (**Figure 7A** and **B**). To elucidate this phenomenon, we evaluated the migration of isolated, positively identified Schwann cells (**Figure 7C**) using a Transwell culture system. The results showed that compared with the WT group, a significantly smaller number of cells in the KO group migrated across the Transwell membrane over 20 hours of culture (**Figure 7D** and **E**). Conversely, primary cultures of WT mouse-derived Schwann cells that were transfected with miR-301a mimics exhibited a significant increase in the number of migrated cells compared with those transfected with NC miRNA (**Figure 7F** and **G**).

### Weakened phagocytosis of Schwann cells with microRNA-301a knockout is restored by microRNA-301a mimics transfection

**Figure 8:**
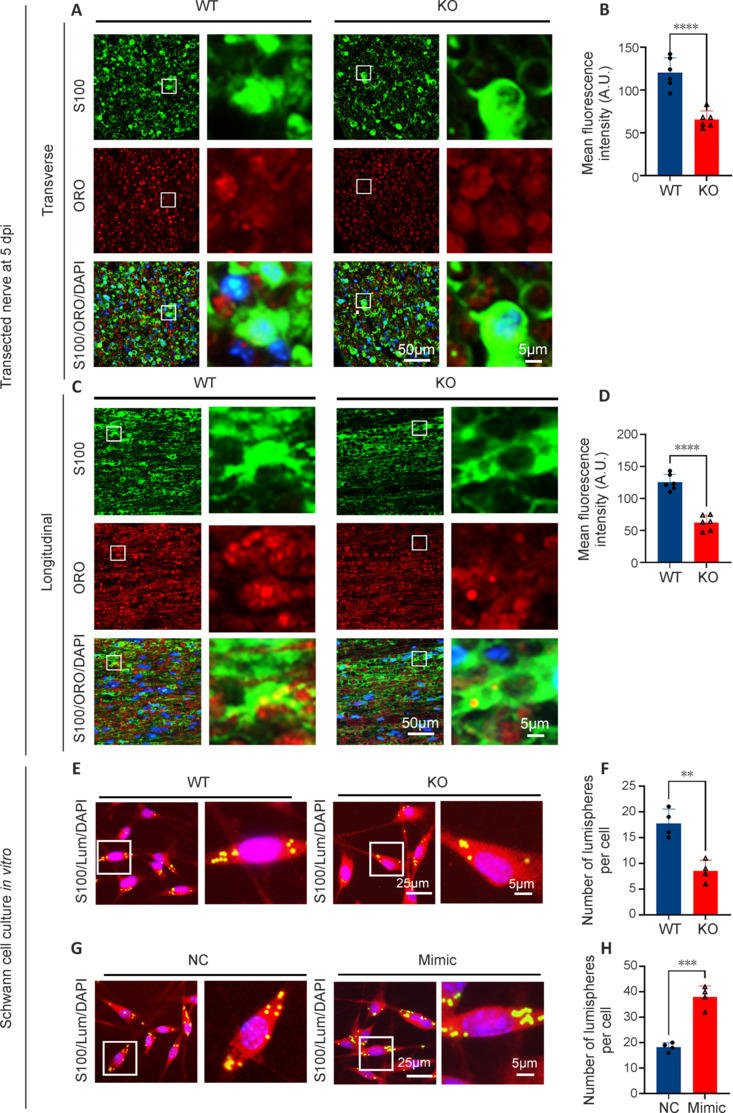
Schwann cell phagocytosis is weakened by miR-301a deficiency and facilitated by transfection with miR-301a mimics. (A, C) Oil red O (ORO) staining (red) and S100 immunofluorescence (green, Alexa Fluor 488) showing lipid droplets engulfed in Schwann cells in transverse sections (A) and longitudinal sections (C) of the transection-injured nerve at 5 days post injury (dpi). (B, D) Quantification analysis showing significantly reduced mean fluorescence intensity of ORO in S100-positive cells in the miR-301a knockout (KO) group compared with that in the wild-type (WT) group (*n* = 6). Counterstaining of nuclei with 4′,6-diamidino-2-phenylindole (DAPI) is also shown. (E, F) S100 immunofluorescence (red; E) and quantification analysis (F) showing a significantly higher number of engulfed lumispheres (Lum; green) in Schwann cells from the WT group than in those from the KO group (*n* = 4). (G, H) S100 immunofluorescence (red; G) and quantification analysis (H) showing a significantly higher number of engulfed lumispheres (green) in Schwann cells from the miR-301a mimics transfection (Mimic) group than in those from the negative control miRNA (NC) group (*n* = 4). Data are expressed as means ± standard deviation; **P < 0.01, ***P < 0.001, ****P < 0.0001 (two-tailed Student’s *t*-test for all data). miR-301a: MicroRNA-301a.

Based on previous studies in macrophages (Xu et al., 2021; Liu et al., 2023), we performed S100 immunofluorescence and ORO staining of transection-injured nerve sections at 5 dpi, and then calculated the ORO-positive fluorescence intensity in each Schwann cell. Quantification analyses of both transverse sections (**Figure 8A** and **B**) and longitudinal sections (**Figure 8C** and **D**) showed that the Schwann cells in the KO group engulfed significantly fewer ORO-positive lipids than those in the WT group. Additionally, the co-culture of Schwann cells with fluorescent microspheres illustrated that cells in the KO group phagocytosed significantly fewer microspheres than those in the WT group (**Figure 8E** and **F**). In contrast, miR-301a mimics-transfected Schwann cells derived from WT mice phagocytosed more fluorescent microspheres than NC-transfected cells (**Figure 8G** and **H**). These results confirmed that miR-301a plays vital roles in the migratory and phagocytic capabilities of Schwann cells.

### MicroRNA-301a may affect Schwann cell functioning through the Yin Yang 1/C-X-C motif receptor 4 pathway

**Figure 9:**
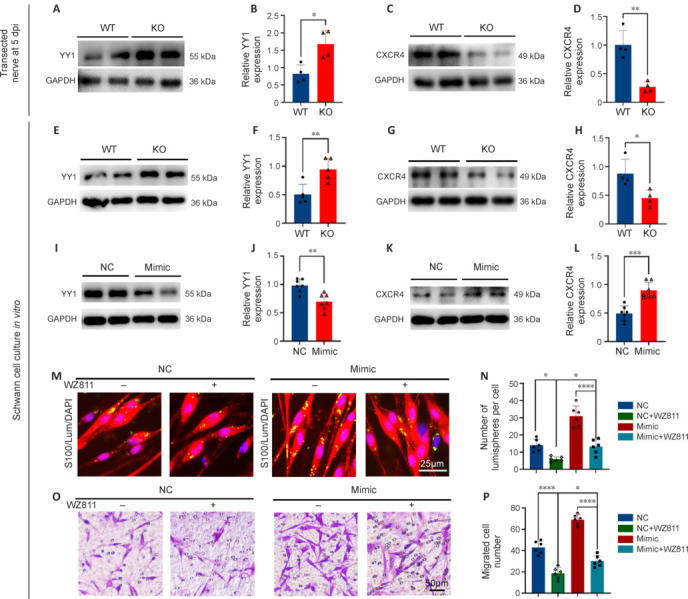
The YY1/CXCR4 pathway is involved in the effects of miR-301a on Schwann cell migration and phagocytosis. (A–D) Western blotting (A, C) and quantification analyses (B, D) showing the protein levels of YY1 and CXCR4 in the transection-injured nerve of wild-type (WT) and miR-301a knockout (KO) mice at 5 days post-injury (dpi) (*n* = 4, two-tailed Student’s *t*-test). (E–H) Western blotting (E, G) and quantification analyses (F, H) showing the protein levels of YY1 (*n* = 5) and CXCR4 (*n* = 4) in WT- and KO-derived Schwann cells (two-tailed Student’s *t*-test). (I–L) Western blotting (I, K) and quantification analyses (J, L) showing the protein levels of YY1 and CXCR4 in negative control miRNA (NC)- and miR-301a mimics (Mimic)-transfected Schwann cells (*n* = 7, two-tailed Student’s *t*-test). (M, N) Representative immunofluorescence images (M) and quantification analysis (N) showing the significantly reduced engulfment of S100-labeled lumispheres (red, Alexa Fluor 568) by Schwann cells in the NC group compared with that in the Mimic group, and the reversal of this trend in transfected cells treated with WZ811 (*n* = 6, one-way analysis of variance followed by the Bonferroni’s multiple comparison test). (O, P) Representative Transwell assay images (O) and quantification analysis (P) showing significantly less migration of Schwann cells in the NC group compared with that in the Mimic group, and reversal of this trend in transfected cells treated with WZ811 (*n* = 6, one-way analysis of variance followed by the Bonferroni’s multiple comparison test). Data expressed as means ± standard deviation; *P < 0.05, **P < 0.01, ****P < 0.001, ****P < 0.0001. CXCR4: C–X–C motif receptor 4; GAPDH: glyceraldehyde-3-phosphate dehydrogenase; miR-301a: microRNA-301a; YY1: *Yin Yang* 1.

To determine whether miR-301a exerts its effects on Schwann cell functioning through the Yin Yang-1 (YY1)/CXCR4 pathway, we examined the expression levels of YY1 and CXCR4 in transection-injured nerves and cultured Schwann cells from miR-301a KO and WT mice. Western blotting results indicated that the miR-301a KO increased YY1 expression (**Figure 9A** and **B**) and downregulated CXCR4 expression (**Figure 9C** and **D**) in transection-injured nerves at 5 dpi. These changes were also observed *in vitro* in cultured Schwann cells isolated from miR-301a-KO mice and WT mice (**Figure 9E–H**). To confirm these results, we transfected the cultured Schwann cells derived from WT mice with miR-301a mimics, which resulted in downregulation of YY1 (**Figure 9I** and **J**) and increased expression of CXCR4 (**Figure 9K** and **L**). Furthermore, treatment of the transfected Schwann cells with the CXCR4-specific inhibitor WZ811 remarkably reversed the effects of miR-301a mimics on phagocytosis, as indexed by lumisphere engulfment (**Figure 9M** and **N**), and migration, as revealed by the Transwell assay (**Figure 9O** and **P**). These results indicated that miR-301a impacts Schwann cell functioning through the YY1/CXCR4 pathway.

## Discussion

Previous studies have reported more than 20 miR-301a target genes in various cells, some of which are well-known regulators of neurodevelopment (Chen et al., 2016; Tavakolpour et al., 2018; Li et al., 2019b). For example, phosphatase and tensin homolog deleted on chromosome ten (PTEN) plays a key role in the development of both the peripheral and central nervous systems (Park et al., 2008; Feng et al., 2019). In addition, PIAS3 and SMAD4 affect neural development through the signal transducer and activator of transcription 3 (STAT3) signaling pathway (Zhang et al., 2017; Zhong et al., 2018). Therefore, we initially speculated that KO of miR-301a would affect nerve development. However, in the present study, the miR-301a KO did not result in significant abnormalities in the structure or function of intact sciatic nerves of mice. Although this result was unexpected, it meant that miR-301a-KO mice could serve as a suitable model for studying the role of miR-301a in nerve injury and repair. Mechanically, we reason that this phenomenon is attributable to the very low expression level of miR-301a in intact nerves, which indicate that miR-301a does not play a role in maintaining the structure and function of intact nerves. Interestingly, we found that the expression level of miR-301a in the sciatic nerve increased sharply after injury, indicating that miR-301a might be involved in the processes of nerve injury and repair and inspiring this study of peripheral nerve regeneration in miR-301a-KO mice. Combining data from a series of morphological, molecular, and functional assessments, we have demonstrated for the first time that miR-301a KO can delay the morphological and functional repair of a crush-injured nerve.

The most important components of the peripheral nerves are neuron-derived axons and Schwann cells, which represent a distinct type of glial cell that myelinates a single axon segment. Considering that our study showed no difference in neurite outgrowth between miR-301a-KO and WT DRG neurons, we found it unlikely that miR-301a impacts nerve repair via regulation of the intrinsic neuronal capability of axonal regeneration. Therefore, subsequent experiments were focused on the biofunctions of Schwann cells. Our previous study demonstrated that KO of miR-301a attenuated the migration and phagocytosis of macrophages (Xu et al., 2022), and elucidated the involvement of both macrophages and Schwann cells in debris clearance and WD progress following peripheral nerve injury. Therefore, the results obtained from the *in vivo* WD model were not conclusive regarding the participation of Schwann cells in this event. To clarify this issue, we prepared an *in vitro* WD model of nerve explants that excluded the recruitment of macrophages into the injured nerve, thereby allowing WD to progress mainly via Schwann cells (Brosius Lutz et al., 2017; Xu et al., 2021). As expected, our findings from this model indicated that miR-301a KO delayed WD *in vitro* just as it did *in vivo*, leading us to propose that miR-301a KO delays WD through effects on both macrophages and Schwann cells. Subsequently, we demonstrated that miR-301a KO weakened the migratory and phagocytic capabilities of Schwann cells through the YY1/CXCR4 pathway, similar to its role in macrophages.

To our knowledge, this is the first study to use both *in vivo* and *in vitro* data to elucidate the role of miR-301a in biofunctions of Schwann cells. The present data suggest that miR-301a plays a vital role in peripheral nerve regeneration, regulating Schwann cell biofunctions via the YY1/CXCR4 pathway. Because many studies have suggested miR-301a inhibition as a prospective strategy for tumor therapy (Yue et al., 2016; Wu et al., 2017; Granda-Díaz et al., 2023), we must caution the future users of miR-301a inhibitors about their side effects on nerve regeneration. If an miR-301a inhibitor cannot be delivered directly to the tumor, it should be avoided for use in cancer patients with nerve damage.

There is one main limitation of this study: we did not utilize Schwann cell-specific miR-301a-KO mice to exclude the effects of miR-301a KO on other cell types. Nevertheless, we conclude that miR-301a upregulation in the injured peripheral nerve is essential for repair of the nerve. Our findings indicating that miR-301a impacts Schwann cell biofunctions via the YY1/CXCR4 pathway to regulate peripheral nerve regeneration warrant further study.

## Additional files:

***Additional Table 1:*** *Antibody list.*

***Additional file 1:*** *Open peer review report 1.*

OPEN PEER REVIEW REPORT 1

## Data Availability

*All data relevant to the study are included in the article or uploaded as Additional files*.
