# Supplementary material for: MicroRNA-301a knockout attenuates peripheral nerve regeneration by delaying Wallerian degeneration
Source: Neural Regen Res. 2024 Jul 29;21(6):2580–9. doi: 10.4103/NRR.NRR-D-24-00081 (PMC13211778; doi:10.4103/NRR.NRR-D-24-00081)
Supplement: Supplementary file 1 [file NRR-21-2580_Suppl1.pdf]

OPEN PEER REVIEW REPORT 1

Name of journal: Neural Regeneration Research
Manuscript NO: NRR-D-24-00081
Title: MiR-301a knockout attenuates the peripheral nerve regeneration via delaying Wallerian degeneration
Reviewer's Name: Bo Hu
Reviewer's country: USA

COMMENTS TO AUTHORS

Strengths:

1. The study makes a significant contribution by elucidating the role of miR-301a in peripheral nerve injury and repair, an area that has not been extensively explored previously.
2. Identifying the YY1/CXCR4 pathway as a potential mechanism for the effects of miR-301a offers a valuable avenue for further research, potentially leading to new therapeutic targets.
3. The use of miR-301a mimics to reverse the effects observed in knockout models strengthens the argument for the specific role of miR-301a, showcasing the study's methodological rigor.

Weaknesses:

1. Not specifying the gender of the animals used in the study is a significant oversight, as gender can influence biological responses and experimental outcomes.
2. The varying number of animals used in different experiments without justification via power calculations raises questions about the statistical robustness of the findings.
3. Poor readability due to language issues can hinder the understanding and dissemination of the research findings.
4. The discussion section focuses heavily on reiterating results rather than providing a comprehensive analysis of the implications and potential future directions of the research.

The manuscript provides valuable insights into the role of miR-301a in the context of peripheral nerve injury and the subsequent repair processes. The authors have demonstrated the role of miR-301a in modulating Schwann cell behavior, which is crucial for the Wallerian degeneration (WD) process. The study's findings on the delay in nerve regeneration upon miR-301a knockout and the reversal of these effects with miR-301a mimic treatment are particularly noteworthy and open new avenues for therapeutic interventions.

However, to enhance the robustness and clarity of the study, the following suggestions are recommended:

1. The gender of the animals used in the study should be clearly stated to assess any potential sex-dependent variations in the experimental outcomes.
2. The study employs varying numbers of animals across different experiments. It would be beneficial to include power calculations to justify these sample sizes and strengthen the statistical validity of the findings.
3. To fully understand the impact of miR-301a knockout on nerve regeneration, it would be beneficial to include morphological data (such as myelin thickness and G ratio) from before the injury. This would provide a clearer baseline for comparison.
4. Including miR-301a expression levels in the knockout nerves within Figure 1i would validate the specificity of the qPCR results. Additionally, providing the primer sequences used for miR-301a in the qPCR experiments would enhance the reproducibility of the study.
5. The manuscript's readability could be improved. It is advisable to have the manuscript reviewed and revised by a professional with expertise in scientific writing to ensure clarity and coherence.
6. Considering the complexity of nerve regeneration, it would be valuable to investigate whether

miR-301a also regulates other cell types involved in the process, such as axons and fibroblasts.
7. Introducing the YY1/CXCR4 pathway in the manuscript's introduction could provide readers with early insight into its proposed significance. Further investigation into the in vivo relevance of this pathway in the context of miR-301a's role would be valuable.
8. In general, their discussion appears to be primarily repetitive of their results while discussing the impact of this work on the field only minimally. Thus, it would be helpful to expand that aspect of the discussion.
